# Glycogen synthase kinase-3 beta (GSK3β)-mediated phosphorylation of ETS1 promotes progression of ovarian carcinoma

**DOI:** 10.18632/aging.202966

**Published:** 2021-05-23

**Authors:** Chia-Lung Tsai, Shih-Ming Jung, Lang-Ming Chi, Chi-Neu Tsai, Chiao-Yun Lin, Angel Chao, Yun-Shien Lee

**Affiliations:** 1 Genomic Medicine Core Laboratory, Chang Gung Memorial Hospital, Taoyuan, Taiwan; 2 Department of Pathology, Chang Gung Memorial Hospital, Linkou Medical Center, and Chang Gung University, Taoyuan, Taiwan; 3 Clinical Proteomics Core Laboratory, Chang Gung Memorial Hospital, Taoyuan, Taiwan; 4 Graduate Institute of Clinical Medical Science, Chang-Gung University, Taoyuan, Taiwan; 5 Department of Surgery, Chang Gung Memorial Hospital, Taoyuan, Taiwan; 6 Gynecologic Cancer Research Center, Chang Gung Memorial Hospital, Taoyuan, Taiwan; 7 Department of Obstetrics and Gynecology, Chang Gung Memorial Hospital, Linkou Medical Center, and Chang Gung University, Taoyuan, Taiwan; 8 Department of Biotechnology, Ming Chuan University, Taoyuan, Taiwan

**Keywords:** GSK3β, ETS1, MMP-9, ovarian cancer, immunohistochemistry

## Abstract

ETS1 − an evolutionarily conserved transcription factor involved in the regulation of a number of cellular processes − is overexpressed in several malignancies, including ovarian cancer. Most studies on ETS1 expression have been focused on the transcriptional and RNA levels, with post-translational control mechanisms remaining relatively unexplored in the pathogenesis of malignancies. Here, we show that ETS1 forms a complex with glycogen synthase kinase-3β (GSK3β). Specifically, GSK3β-mediated phosphorylation of ETS1 at threonine 265 and serine 269 promoted protein stability, induced the transcriptional activation of matrix metalloproteinase (*MMP*)*-9*, and increased cell migration. *In vivo* experiments revealed that a GSK3β inhibitor was able to suppress both endogenous ETS1 expression and induction of *MMP-9* expression. Upon generation of a specific antibody against phosphorylated ETS1, we demonstrated that phospho-ETS1 immunohistochemical expression in ovarian cancer specimens was correlated with that of MMP-9. Notably, the cumulative overall survival of patients with low phospho-ETS1 histoscores was significantly longer than that of those showing higher scores. We conclude that the GSK3β/ETS1/MMP-9 axis may regulate the biological aggressiveness of ovarian cancer and can serve as a prognostic factor in patients with this malignancy.

## INTRODUCTION

Ovarian cancer is a deadly gynecologic malignancy frequently diagnosed at advanced stages. Primary debulking surgery followed by platinum-based adjuvant chemotherapy represents the mainstay of therapy. Although an initial response to primary treatment can be observed even in advanced-phase disease, recurrences and resistance to subsequent chemotherapy remain common [1]. In this scenario, a better understanding of the molecular mechanisms involved in ovarian tumorigenesis may pave the way to novel targeted treatment strategies for recurrent/advanced disease.

ETS1 belongs to the evolutionarily conserved ETS family of transcription factors [2] involved in the regulation of a number of cellular processes [3]. ETS1 has been found to be overexpressed in several solid tumors, including breast [4], colorectal [5], endometrial [6], lung [7], and ovarian [8] cancers. Moreover, an increased ETS1 expression has been associated with high tumor grading, poor differentiation, and more pronounced invasiveness [3, 9] – ultimately being considered a marker of poor prognosis [10, 11].

Several post-translational modifications – including phosphorylation – can influence ETS1 biological activity. Although phosphorylation by SRC kinase and protein kinase Cα (PKCα) can increase ETS1 stability [12, 13], the opposite effect is observed when phosphorylation is catalyzed by calcium-dependent protein kinase II (CaMKII) [14, 15]. Similarly, the transcriptional activity of ETS1 is activated by ERK1/2 phosphorylation [16] and blocked by CaMKII [14, 15]. When stabilized and activated by kinases, ETS1 promotes cancer cell invasiveness by inducing the expression of matrix metalloproteinases (MMPs) [8, 17]. Accordingly, a previous study conducted in breast cancer has shown that overexpression of ETS1 in the presence of epidermal growth factor (EGF) enhances *MMP-9* promoter activity [18]. A strict association between ETS1 and MMP expression levels has been consistently demonstrated in ovarian and lung malignancies [8, 19].

MMPs are zinc metalloenzymes that digest extracellular matrix proteins – ultimately playing a key role in cancer growth, invasion, and metastasis [20]. Specifically, upregulation of MMP-9 has been associated with the progression of several malignancies [21] – with its expression levels being positively regulated by numerous transcription factors, including NF-κB [22], AP1 [23], and ETS1 [24]. ETS1 is a helix-loop-helix protein capable of binding to the GGAA/T core motif in the *MMP-9* promoter region [25]. Interestingly, the co-expression of ETS1 and MMP-9 portends a poor prognosis in solid malignancies [17, 26].

Glycogen synthase kinase-3β (GSK3β) is a serine/threonine protein kinase that regulates several cellular processes – including proliferation, differentiation, and migration [27]. GSK3β substrates – which are characterized by the presence of a common specific phosphoryl protein motif-(S/T)XXX(S/T) – play a fundamental regulatory role in cell migration and invasiveness [28]. As a consequence, there is a growing interest in GSK3β as a therapeutic target in cancer [29].

In this study, we provide evidence that GSK3β phosphorylates ETS1 at two specific sites (threonine 265 and serine 269) that are part of the GSK3β phosphorylation motif. Site-directed mutagenesis of these residues reduced protein stability, *MMP-9* transcriptional activation, and cell migration. The effect of a GSK3β inhibitor on endogenous ETS1 expression and induction of *MMP-9* was investigated using *in vivo* experiments. Finally, we generated a specific antibody against phosphorylated ETS1 and examined its expression levels in human ovarian cancer specimens. Taken together, our data may shed more light into the role of the GSK3β/ETS1/MMP-9 axis in ovarian tumorigenesis – potentially paving the way for novel therapeutics.

## RESULTS

### ETS1 interacts with GSK3β

**Figure 1:**
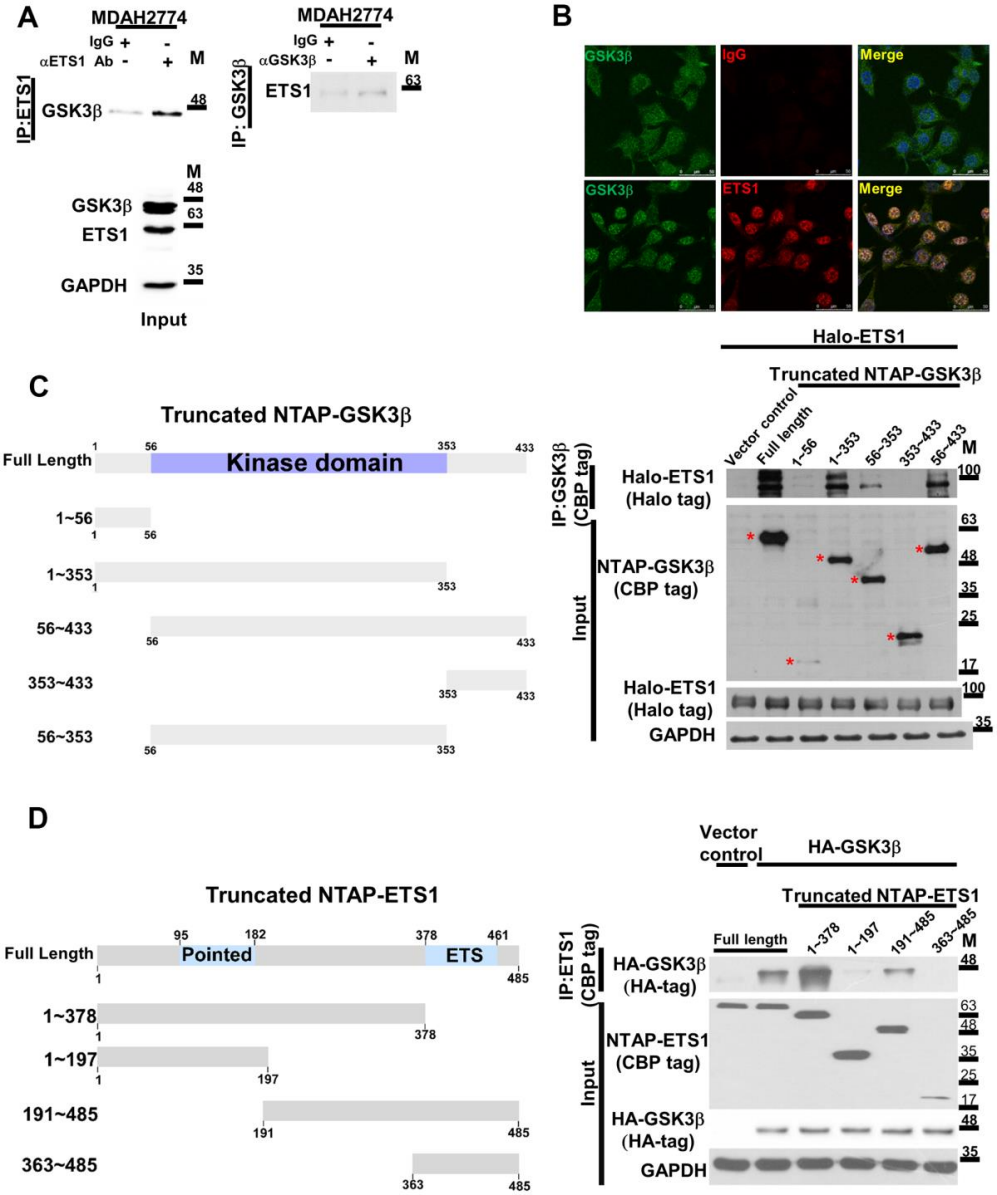
**The GSK3β phosphorylation complex includes ETS1.** (**A**) The endogenous GSK3β-ETS1 complex was pulled-down in ovarian cancer cell lysates (MDAH2774) using an anti-ETS1 antibody (left panel), an anti-GSK3β antibody (right panel), and a control IgG. The associated GSK3β protein (ETS1 antibody pull-down, left panel) or ETS1 (GSK3β antibody pull-down, right panel) were determined by western blot. Levels of endogenous GSK3β and ETS1 are shown in the lower panel. (**B**) Confocal microscopy revealed the presence of a GSK3β-ETS1 complex in MDAH2774 cells. Cells were double-stained with the GSK3β antibody (green signal, left panel), the ETS1 antibody (red signal, middle lower panel), or the control IgG (middle upper panel). Two proteins showed colocalization (yellow signal) as revealed by overlapping green and red signals (right panel). (**C**) 293 cells were transfected with full-length ETS1 (Halo-ETS1) and control vector, the full length and truncated GSK3β constructs (CBP tag). The presence of ETS1 aggregated in a complex was examined with an anti-Halo tag antibody. Asterisks indicate the full length and truncated GSK3β protein. The reference number of amino acid sequence for GSK3β isoform 1 is NP_002084.2 while the nucleotide number is NM_002093.4. (**D**) Full-length GSK3β (HA-GSK3β) or control vector was co-transfected with the full length and truncated ETS1 constructs (FL: full length, 1−378, 1−197, 191−486, and 363−486) into 293 cells. Upon purification with streptavidin beads, co-precipitated GSK3β was detected using an anti-HA antibody. The reference number of amino acid sequence for ETS1 is NP_001137292.1.

The potential interaction between ETS1 and GSK3β was investigated by means of immunoprecipitation. After pulling-down of endogenous ETS1 with a specific antibody, endogenous GSK3β was identified. Notably, we also observed an endogenous ETS1-GSK3β complex in ovarian cancer cell line (Figure 1A). Co-localization of the two proteins within cells was confirmed by confocal microscopy (Figure 1B). Serial truncated GSK3β and ETS1 constructs were used to map the interaction domains of ETS1 and GSK3β. The kinase domain of GSK3β (56-353/GSK3β) was found to be critical for the ETS1 interaction (Figure 1C). Interestingly, deletion of the central ETS1 region (1-197/ETS1 and 363−486/ETS1) reduced its interaction with GSK3β (Figure 1D). Altogether, these data indicate that 1) the kinase domain of GSK3β is responsible of its interaction with ETS1, 2) the central region of ETS1 (197−363) is necessary for its interaction with GSK3β.

### ETS1 is a GSK3β substrate

**Figure 2:**
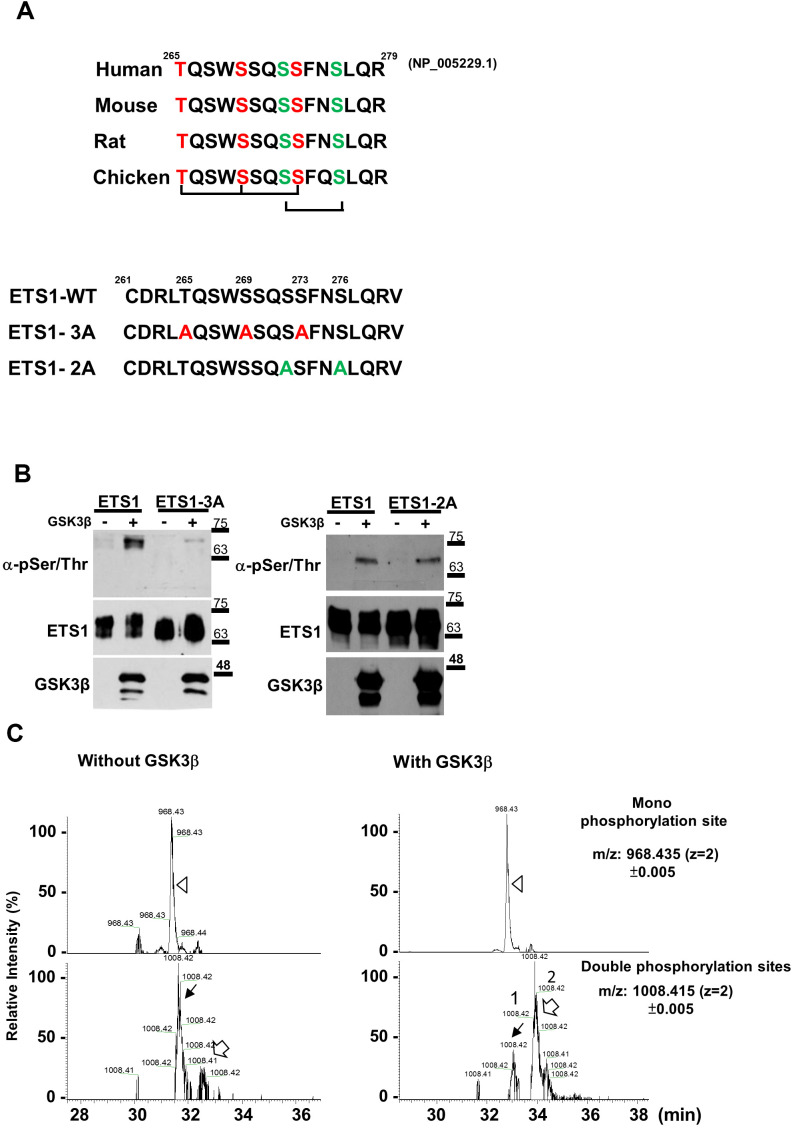
**GSK3β phosphorylates ETS1 protein.** (**A**) The ETS1 protein is characterized by the presence of conserved GSK3β phosphorylation motifs in different species (human, mouse, rat, and chicken). Two potential motifs (highlighted in red and green letters) underwent mutagenesis and were termed ETS1-3A (red letters) and ETS1-2A (green letters). (**B**) *In vitro* kinase assay. ETS1, ETS1-3A, and ETS1-2A were transfected into 293 cells and subsequently purified using a halo resin. After incubation with GSK3β and ATP, phosphorylated ETS1 was detected using anti-threonine/serine antibody. (**C**) Phosphorylated peptides of the ETS1 protein were subjected to an *in vitro* kinase reaction either with or without GSK3β following enrichment with TiO_2_ beads. Subsequently, an LC-MS/MS analysis was performed. The ion chromatograms of the peptide potentially targeted by GSK3β (LTQSWSSQSSFNSLQR at amino acids 264-279 of the ETS1 protein) with single (968.435±0.005 m/z, upper panel, white triangle) and double (1008.415±0.005 m/z, lower panel, black and white arrows) phosphorylation sites were extracted. Two double phosphorylation events were identified – with a relatively marked difference with and without GSK3β treatment.

ETS1 has two potential GSK3β phosphorylation motifs (highlighted in red letters T265/S269/S273, and green letters, S272/S276, Figure 2A) [28]. To identify the GSK3β phosphorylation motifs in ETS1, we generated wild-type, triple mutant (ETS1-3A), and double mutant (ETS1-2A) ETS1 (Figure 2B) – which were subsequently purified and incubated with GSK3β and ATP. After probing with the anti-phospho-serine/threonine antibody on a SDS-denatured membrane, we found lower phospho-serine/threonine ETS1-3A levels compared with wild-type – although the extent of phosphorylation was similar for the ETS1-2A protein (Figure 2B).

To identify the amino acid residues phosphorylated by GSK3β, the wild-type ETS1 products of the *in vitro* kinase assay were digested with trypsin and subjected to phosphosphopeptide enrichment for LC-MS/MS. The mono- and double-phosphorylation events of the ETS1 tryptic peptide (LTQSWSSQSSFNSLQR) were determined by LC-MS/MS. The extracted ion chromatography spectra are shown in Figure 2C. There were no differences in the monophsophorylation site forms of ETS1 (upper panel, Figure 2C). However, there were two distinct double-phosphorylated forms of the ETS1 tryptic peptide – which were denoted by black (peak1) and white (peak2) arrows in Figure 2C (lower panel). When the relative intensities of the two double-phosphorylated peptide forms were analyzed with and without GSK3β treatment, the signal of peak2 double-phosphorylated peptide was found to be markedly increased upon GSK3β exposure. Two different double-phosphorylated isoforms can be targeted by GSK3β (Figure 2B). Therefore, two synthetic double-phosphorylated peptides (pT2pS6, also known as T265/S269 and pS6pS10, also known as S272/S276) were generated to identify the exact phospho-amino acid residues in the GSK3β-enhanced ETS1 phosphopeptide (peak2). As shown in Supplementary Figure 1A, 1B, the fragmentation profile (MS/MS spectra) of the peak2 precursor ion (m/z 1008.41, z = 2, upper panel) showed non-phosphorylation signals in the y7 and y8 fragments (m/z 851.44 and 938.47, respectively). Notably, the corresponding masses were identical (y7, m/z 851.43; y8, 938.47) to those observed in the pT2pS6 spectrum profile (middle panel) but were different from those of the pS6pS10 peptide fragmentation spectra (lower panel). These results clearly indicate that phosphorylation on the first threonine and the second serine residues of the ETS1 peptide occurs only upon GSK3β treatment. To further confirm the presence and the role of this double-phosphorylated form, the pT2pS6 synthetic peptide was used as an immunogen to generate a polyclonal antibody capable of detecting this specific phospho-ETS1 isoform in cell lines and tissue specimens.

### GSK3β phosphorylates ETS1 to maintain protein stability

**Figure 3:**
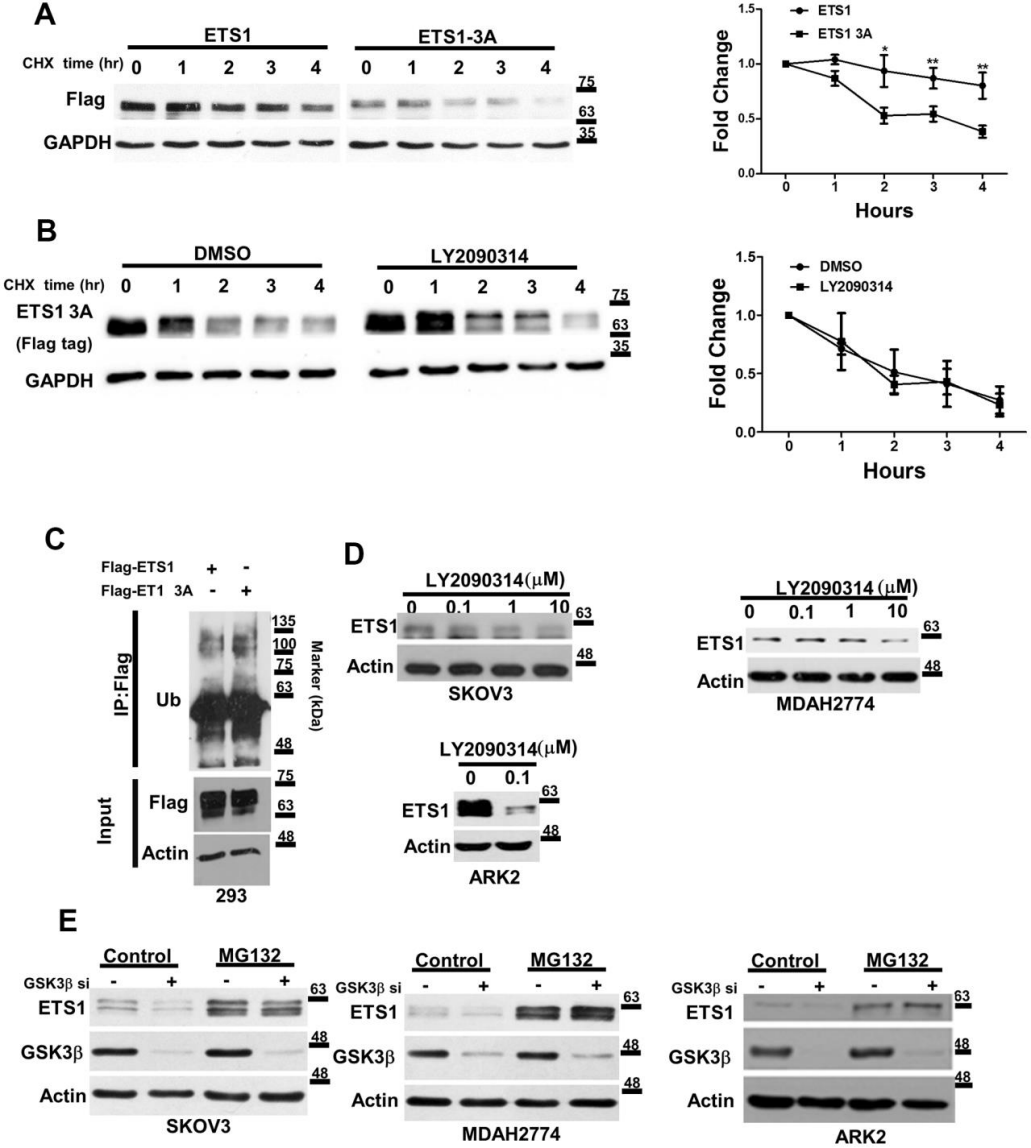
**GSK3β regulates the stability of ETS1.** (**A**) Flag-ETS1 and Flag-ETS1-3A were overexpressed in 293 cells and harvested at the reported time points in presence of cycloheximide (CHX). Flag-ETS1 and Flag-ETS1-3A protein levels were analyzed using an anti-Flag antibody. Endogenous GAPDH was used as a loading control, followed by normalization with an anti-GAPDH antibody. Fold changes in Flag-ETS1 or Flag-ETS1-3A protein levels are plotted in the right panel. Results are means ± standard errors of the mean from three independent experiments. *p < 0.05. (**B**) Flag-ETS1-3A were overexpressed in 293 cells and pre-treated with vehicle control or LY2090314 (0.1μM) for 1 h, finally harvested at the reported time points in the presence of cycloheximide (CHX). (**C**) Flag-ETS1 and Flag-ETS1-3A were overexpressed in 293 cells and harvested after treatment with MG132 (10 μM) for 5 h. Flag-ETS1 and Flag-ETS1-3A were precipitated with an anti-Flag antibody, whereas ubiquitin-bound proteins were detected with an anti-ubiquitin antibody. An Anti-Flag served as an input control and specific antibodies were used to detect Flag-ETS1, Flag-ETS1-3A, and actin. (**D**) The specific GSK3β inhibitor LY2090314 promoted ETS1 protein degradation. Ovarian cancer cells (SKOV3 and MDAH2774) and endometrial cancer cells (ARK2) were starved in OPTI-MEM medium in presence of the reported LY2090314 concentration for 24 h. Endogenous ETS1 and actin were analyzed with western blot using specific antibodies. (**E**) Ovarian and endometrial cancer cells were transfected with GSK3β siRNA for 72 h, and subsequently treated with MG132 (10 μM) or a vehicle for 5 h. ETS1, GSK3β, and actin protein levels were examined with western blot.

To investigate the impact of GSK3β-mediated ETS1 phosphorylation, we initially determined the protein turnover rate in 293 cells overexpressing wild-type ETS1 and ETS1-3A in presence of cycloheximide (in order to inhibit protein synthesis). Degradation of ETS1-3A occurred faster than that of wild-type ETS1 (Figure 3A). Furthermore, GSK3β inhibitor (LY2090314) did not alter the stability of ETS1 (Figure 3B). Because conjugation of ubiquitin with proteins plays a key role in protein breakdown, ETS1 and ETS1-3A were pulled-down and probed with an anti-ubiquitin antibody. Ubiquitin levels were then assayed in the presence of the proteasome inhibitor-MG132. The results revealed that levels of ubiquitin-labeled ETS1-3A were higher than those of wild-type ETS1 (Figure 3C). Moreover, the GSK3β inhibitor LY2090314 – which has already undergone clinical testing [30]– induced ETS1 degradation in ovarian and endometrial cancer cells (Figure 3D), an effect blocked by MG132 in the presence of GSK3β siRNA (Figure 3E). Taken together, these data suggest that ETS1 phosphorylation by GSK3β regulates its stability.

### GSK3β phosphorylates ETS1 to regulate MMP-9 expression

**Figure 4:**
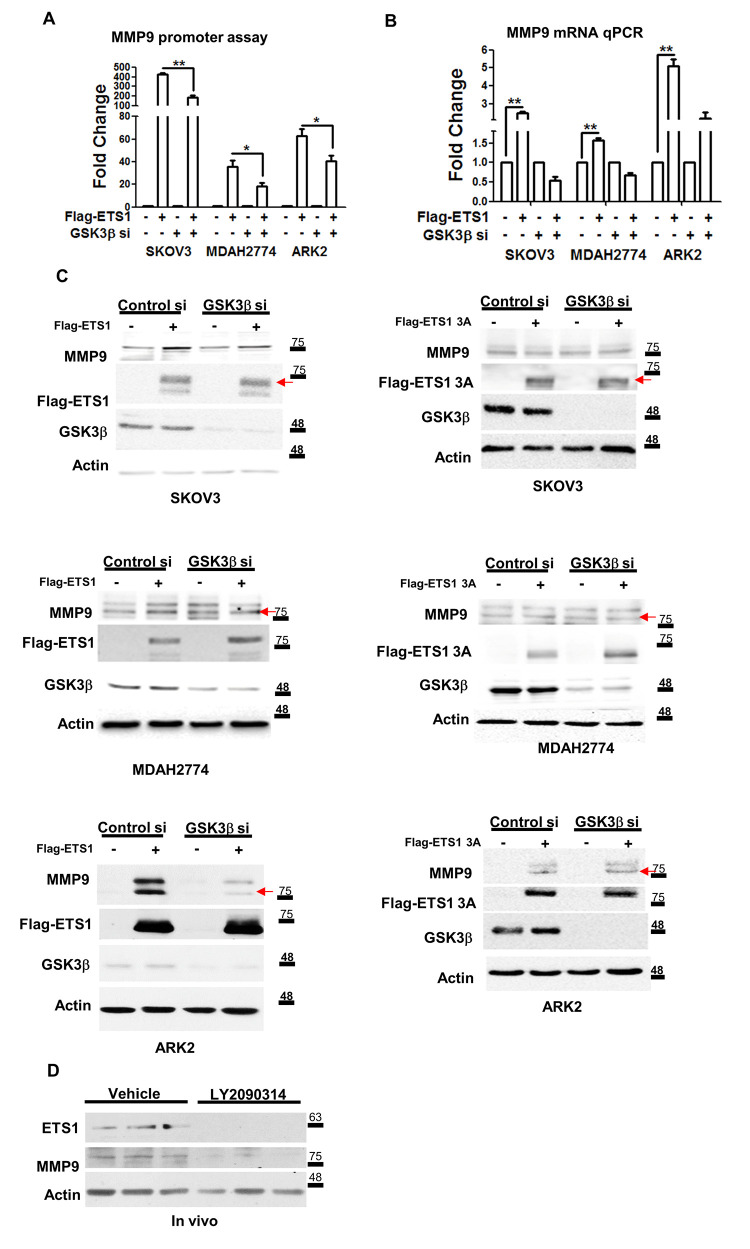
**ETS1 activates MMP-9 expression in presence of GSK3β.** (**A**) The *MMP-9* promoter-luciferase construct was co-transfected with a control vector (labeled as “-” at the bottom of the figure) and Flag-ETS1 in presence or absence of the GSK3β si in SKOV3, MDAH2774 and ARK2cells. (**B**) *MMP-9* mRNA was detected with qPCR in overexpressing Flag-ETS1 or control vector cells in presence or absence of the siGSK3β. The *MMP-9* promoter activity (**A**) and qPCR results (**B**) in cells transfected with the control vector (Flag-control and si-control) cells were set at 1. Results shown are the mean ± standard errors of the mean from three independent experiments. (**C**) Induction of MMP-9 by Flag-ETS1 (left panel) or Flag-ETS1 3A (right panel), was suppressed in cells in which the GSK3β expression was knocked-down for 72 h, and subsequently treated with MG132 (10 μM) for 5 h. The red arrows indicate the Flag-ETS1, Flag-ETS13A and mature MMP9. (**D**) ETS1 and MMP-9 expression in tumors from mice treated with vehicle or LY2090314 (10 mg/kg) twice a week for four weeks. Protein levels of ETS1, MMP-9, and actin were determined by western blot. Asterisks denote statistical significance (*p < 0.05, **p < 0.01).

Because *MMP-9* is one of the ETS1 downstream target genes [18], we investigated the extent to which ETS1 and ETS1-3A promoted *MMP-9* transcriptional activity. First, we analyzed the *MMP-9* promoter to identify ETS1 responsive regions. Overexpression of ETS1 was able to induce both MMP-9 protein and activity in ARK2 cells (Supplementary Figure 2A). After transfection with ETS1 and different truncated *MMP-9* promoter constructs, ETS1 was found to induce a 10-fold increase in promoter activity when the -580 promoter construct was used (Supplementary Figure 2B). All of the subsequent experiments were therefore performed with this construct. To confirm the role of GSK3β in promoting *MMP-9* expression, we examined its induction in cancer cells co-transfected with the ETS1 expression vector and GSK3β siRNA. Silencing of endogenous GSK3β was found to block *MMP-9* promoter activity (Figure 4A), as well as its mRNA (Figure 4B) and protein (Figure 4C) expression. The induction levels of MMP9 in overexpressed ETS1-3A cell lines showed no difference in the absence or presence of GSK3β siRNA (Figure 4C). Because we have previously shown that LY2090314 can inhibit murine ovarian cancer-MOSEC cells growth [31], we also examined the endogenous ETS1 and MMP-9 protein levels in the same experimental tumors of LY2090314-treated mice. LY2090314 was found to inhibit both ETS1 and MMP-9 protein expression (Figure 4D).

**Figure 5:**
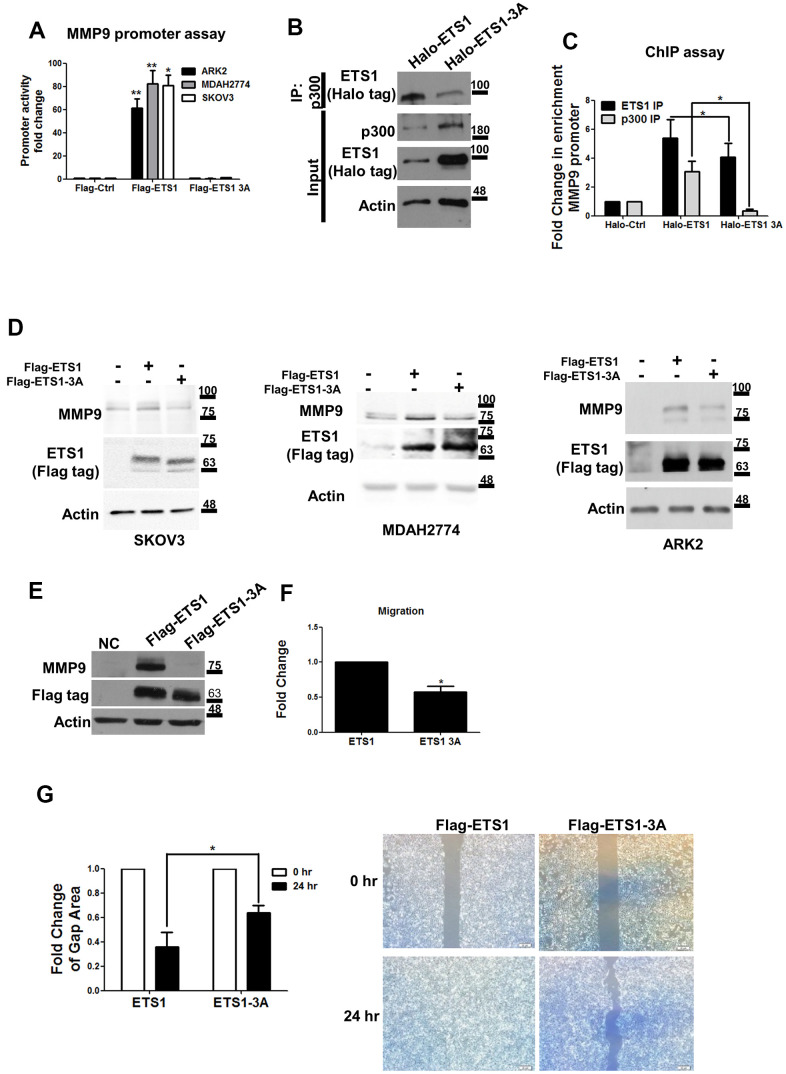
**Mutant ETS1 is unable to activate MMP-9 expression.** (**A**) *MMP-9* promoter luciferase assays were co-transfected with the *MMP-9* promoter construct using Flag-control, Flag-ETS1 or Flag-ETS1-3A in ARK2 (black box), MDAH2774 (gray box), and SKOV3 (white box) cells 48 h later. The *MMP-9* promoter activity in transfected Flag-control cells was set as 1. Results are means ± standard errors of the mean from three independent experiments. (**B**) An anti-p300 antibody was able to precipitate Halo-ETS1 or Halo-ETS1-3A in overexpressing 293 cells. Halo-ETS1 or Halo-ETS1-3A in association with p300 was identified with an anti-Halo tag antibody. (**C**) ChIP assays were analyzed with Halo-ETS1, Halo-ETS1-3A, and endogenous p300 on the *MMP-9* promoter in overexpressing 293 cells. Chromatin DNA was crosslinked with Halo-ETS1 or Halo-ETS1-3A and subsequently purified with a Halo-tag resin. Associated p300 was precipitated with an anti-p300 antibody. Enrichment of the *MMP-9* promoter was identified by qPCR. Results are means ± standard errors of the mean from three independent experiments. (**D**) MMP-9 protein levels were increased by Halo-ETS1 or Halo-ETS1-3A in SKOV3, MDAH2774 and ARK2 cells. (**E**) MMP-9 expression was detected in 293 cells stably transfected Flag-ETS1. (**F**) The results of the transwell migration assay revealed that the migration ability of cells expressing wild-type ETS1 was higher than that of cells expressing ETS1-3A. Quantification of cell migration ability was performed by direct counting. (**G**) In wound closure assay, the cell-free gap area was quantified with the image J software (left panel). Cells characterized by higher migration ability produced smaller cell-free gap areas. Cell-free gap areas at 0 and 24 h are shown as open and black bars, respectively. Results in (**F**, **G**) are means ± standard errors of the mean from three independent experiments. Asterisks denote statistical significance (*p < 0.05, **p < 0.01).

Furthermore, wild-type ETS1, but not ETS1-3A, was able to induce *MMP-9* promoter activity in ARK2, MDAH2774, and SKOV3 cells (Figure 5A). p300 is a histone acetyltransferase which is able to bind ETS1 to serve as a transcription coactivator [32, 33]. To shed more light on the potential role of p300 in ETS1-mediated *MMP-9* regulation, the interaction of p300 with ETS1 and ETS1-3A was examined. ETS1-3A was found to interact with p300 at a lesser extent than ETS1 (Figure 5B). In addition, ETS1-3A had a lower capacity to bind the *MMP-9* promoter (*MMP9* promoter -166 region in Supplementary Figure 2B) compared with ETS1. Results of chromatin immunoprecipitation experiments revealed that a lower amount of p300 was recruited by ETS-3A on the *MMP-9* promoter region (Figure 5C). *MMP-9* induction was less prominent in ETS1-3A-overexpressing cancer as compared with that observed when ETS1 was overexpressed (Figure 5D).

Taken together, these results indicate that ETS1 phosphorylation catalyzed by GSK3β can promote its interaction with p300 and induce the expression of *MMP-9* as a downstream target gene.

### GSK3β mediates ETS1 phosphorylation and regulates cell migration

Stable expression of Flag-ETS1 was found to induce MMP-9 protein levels, but not in Flag-ETS1-3A in 293 cells. (Figure 5E). Cell migration ability was tested using the transwell migration assay and the wound healing assay. Flag-ETS1-expressing cells were characterized by a higher cell migration ability compared with Flag-ETS1-expressing 3A cells (Figure 5F). Results of the wound closure assay revealed that the cell-free gap area for Flag-ETS1 was lower than that observed for ETS1-3A (Figure 5G). These findings indicate that the GSK3β/ETS1/MMP-9 axis may regulate tumor cell migration.

### Immunohistochemical expression of phospho-ETS1 Thr265, Ser269, and MMP-9 in human ovarian cancer

Specific polyclonal antibodies against phospho-ETS1 Thr265, Ser269 were generated to investigate its immunohistochemical expression in ovarian cancer tissues. Using cell lysates obtained from the breast cancer cell line MDA-MB-231 as a positive control, the antibody was found to specifically detect phospho-ETS1 Thr265, Ser269 both in the absence and presence of the control peptide (i.e., without phosphorylated residues). However, the signal was no longer evident when the antibody was pre-incubated with the phosphorylated peptide (Supplementary Figure 3A), and downregulated in the purified ETS1 protein from GSK3β inhibitor treated cells (Supplementary Figure 3B). Similar findings were obtained when ovarian cancer tissue was used (Supplementary Figure 3C). These results confirmed the specificity of the anti- phospho-ETS1 Thr265, Ser269 antibody. We subsequently analyzed the expression of phospho-ETS1 in nine different ovarian cancer cell lines. Although all of them were found to express total ETS1, the phospho-ETS1 signal was evident in four cell lines only (TOV112D, OV90, MDAH2774, and SKOV3 cells) in which MMP-9 was expressed as well (Supplementary Figure 4A). Knockdown of endogenous GSK3β in ovarian cancer cells inhibited both phospho-ETS1 and MMP-9 expression (Supplementary Figure 4B). Comparing with SKOV3 and MDAH2774 cells, non-malignant ovarian IOSE-80PC cells and epithelial 293 cells were found to have lower MMP9 and phospho-ETS1 expression. However, higher ETS1 and GSK3β were expressed in IOSE-80PC cells, suggesting that phosphorylation of ETS1 is mediated via GSK3β thereby activates *MMP9* expression in cancer cells (Supplementary Figure 4C).

**Figure 6:**
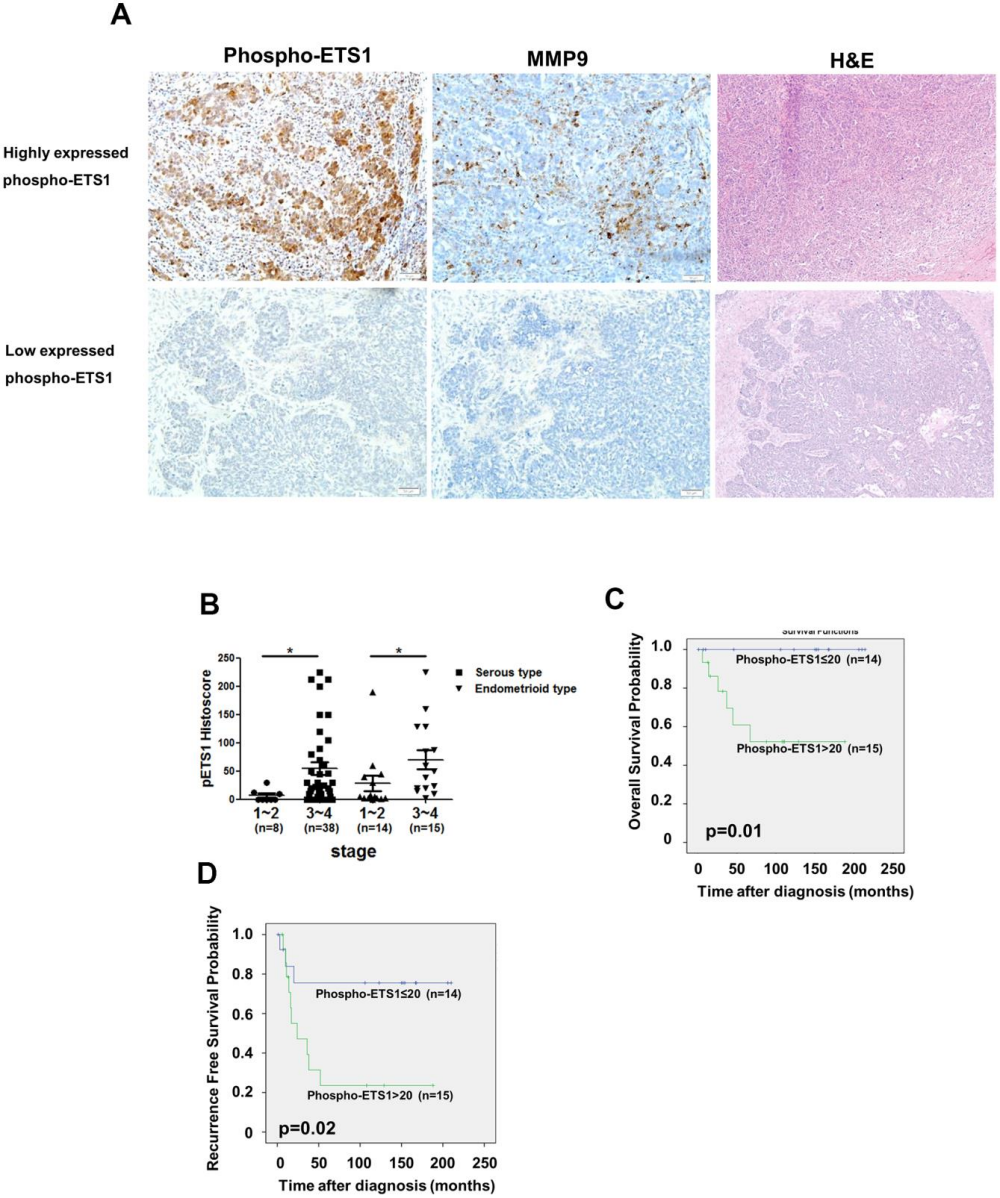
**Phospho-ETS1 expression is associated with tumor stage.** (**A**) Immunohistochemical staining of phospho-ETS1 and MMP-9 in ovarian cancer tissues. Tissue with high and low expressed phospho-ETS1 are shown in the upper and lower panels, respectively. (**B**) Advanced-stage malignancies (stages 3−4) showed a higher immunohistochemical expression of phospho-ETS1 as compared with early-stages tumors (stages 1−2) both in serous (black square) and endometrioid (black triangle) ovarian carcinomas. Asterisks in B denote statistical significance (*p < 0.05). Kaplan-Meier plots of overall (**C**) and recurrence-free survival (**D**) in endometrioid type ovarian cancer patients (n = 29) were constructed according to phospho-ETS1 histoscores. The optimal cutoff for phospho-ETS1 histoscore in the prediction of overall survival and recurrence-free survival was 20.

We also analyzed phospho-ETS1 and MMP-9 in ovarian cancer tissues. The results revealed that MMP-9 expression was higher in tissue areas characterized by high phospho-ETS1 expression (Figure 6A, upper panel) as compared with areas showing lower phospho-ETS1 expression (Figure 6A, lower panel). Moreover, advanced serous and endometrioid ovarian malignancies were characterized by a high phospho-ETS1 expression (reflecting the results obtained in SKOV3 and MDAH2774 cells, respectively (Figure 6B). Kaplan-Meier analysis revealed that the cumulative overall survival (Figure 6C) and recurrence-free survival (Figure 6D) of patients with a phospho-ETS1 histoscore ≤ 20 were significantly higher than those cases with scores >20 in endometrioid type ovarian cancer – suggesting that it may serve as a prognostic biomarker in this malignancy.

## DISCUSSION

**Figure 7:**
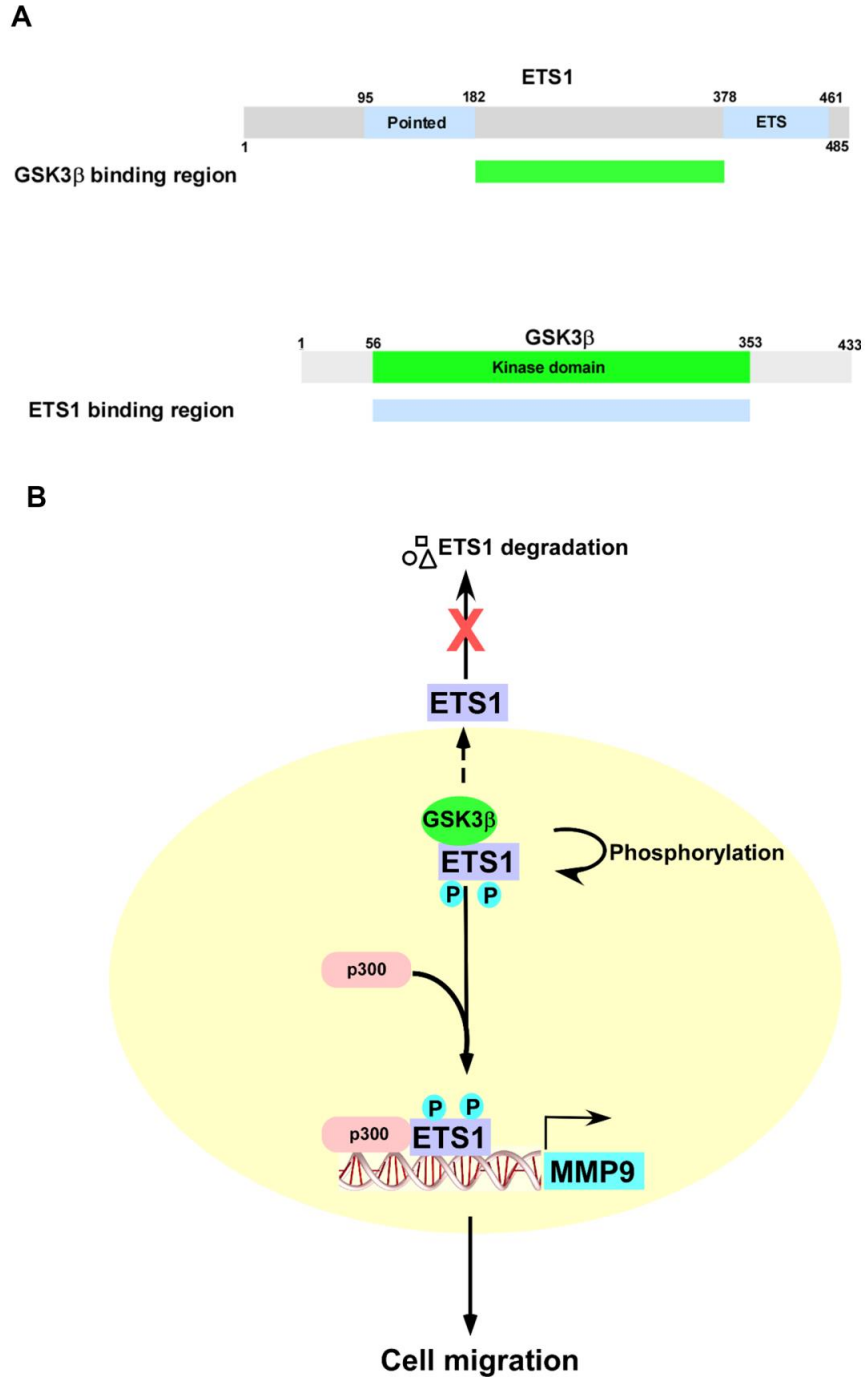
**Schematic representation of the role played by the GSK3β/ETS1/MMP-9 axis in cell migration.** (**A**) Summary of the interactions between different GSK3β and ETS1 domains. (**B**) Upon phosphorylation by GSK3β, ETS1 interacts with p300 to activate the *MMP-9* promoter. Inhibition of GSK3β activity results in ETS1 degradation.

The results of our study indicate that GSK3β phosphorylates ETS1 at T265/S269 and maintains its protein stability. Upon phosphorylation by GSK3β, ETS1 binds the histone acetyltransferase p300 and activates *MMP-9* transcription by acting on its promoter (Figure 7). Notably, the use of LY2090314 – a specific GSK3β inhibitor – was found to inhibit both ETS1 and *MMP-9* expression *in vivo*. Altogether, our data suggest additional roles of GSK3β/ETS1/MMP-9 pathway in ovarian malignancies.

GSK3β is multifunctional kinase that has been implicated in various aspects of tumorigenesis. As a tumor suppressor, GSK3β can phosphorylate and inactivate several oncogenes, including β-catenin, c-Myc, and cyclin D1 – ultimately inhibiting tumor cell proliferation [28]. Previous studies have also shown that GSK3β inhibition can interfere with certain oncogenic pathways – including notch and hedgehog (Hh) [34, 35]. There is also evidence to suggest that the lysine-specific histone demethylase 1A (KDM1A/LSD1) oncoprotein is stabilized by GSK3β-mediated phosphorylation [31, 36]. In addition, clinical studies have shown that GSK3β overexpression portends a poor prognosis in patients with ovarian and lung malignancies [37, 38]. These findings demonstrate that GSK3β may exert distinct – and even opposite – functions in different tissues, disease stages, and tumor types.

GSK3β is also involved in epithelial-mesenchymal transition (EMT) through the transcriptional factor Snail to repress E-cadherin [39]. In addition, it activates *MMP* transcription [40] via phosphorylation-mediated Snail degradation – ultimately preventing EMT [41]. Although this observation may suggest an inverse relationship between GSK3β and MMP protein expression, our immunohistochemical data indicate that both GSK3β and MMP-9 are overexpressed in advanced-stage ovarian cancer [42]. It is conceivable that – in addition to the GSK3β/Snail pathway – the GSK3β/ETS1/MMP-9 axis may be involved in ovarian cancer spread.

In this study, we have also shown that GSK3β-mediated phosphorylation of promoted ETS1 protein stability as well as its transcriptional activity and protein-binding capacity. Compared with wild-type ETS1, ETS1-3A was unable to induce *MMP-9* promoter activity – ultimately preventing MMP-9 protein expression (Figure 5A, 5C). Similar findings were obtained when cancer cells were co-transfected with wild-type ETS1 and the shGSK3β vector (Figure 5). Overexpression of ETS1 was capable of inducing *MMP-9* expression, but this effect was no longer evident when GSK3β was knocked-down. These data indicate that GSK3β regulates ETS1 transcriptional activity and is capable of blocking the expression of ETS1 downstream genes. We also demonstrated that ETS1-3A displayed a reduced capacity to bind both the co-transcriptional activator p300 and the *MMP-9* gene promoter (Figure 5B, 5C).

The ETS1 protein is characterized by the presence of different domains − including a pointed domain, a transactivation domain, and an auto-inhibitory module (which is distinct from the ETS DNA-binding domain) [3]. Importantly, the capacity of ETS1 to bind DNA is regulated by the auto-inhibitory module. Four α-helices of this module are capable of interacting with the ETS DNA-binding domain [3]. Notably, the phosphorylation status of the serine-rich region (SRR, serine residues 251, 257, 282, 285) located in the proximity of the auto-inhibitory module inhibits DNA binding. Phosphorylation of the SRR catalyzed by CaMKII can decrease both DNA binding capacity and ETS1 transcriptional activity [15]. Our results indicate that GSK3β is able to phosphorylate the threonine 265 and serine 269 residues located in the SRR region. Site-directed mutagenesis of these amino acids or GSK3β silencing were found to abrogate ETS1 transcriptional ability, its interaction with p300, as well as its DNA binding capacity (Figure 4, Figure 5A–5C). We therefore speculate that GSK3β and CaMKII can both control the ability of ETS1 to bind DNA, albeit in an opposite manner (i.e., GSK3β promotes and CAMKII abrogates ETS1 binding to DNA). To our knowledge, this is the first comprehensive description of how two different kinases can regulate ETS1 biological activity.

We conclude that the GSK3β/ETS1/MMP-9 axis regulates the biological aggressiveness of ovarian cancer. Specifically, we demonstrated that 1) GSK3β-mediated phosphorylation of ETS1 regulates its protein stability and transcriptional activity and 2) the expression levels of phospho-ETS1 are associated with ovarian cancer stage and can be used as a biomarker to predict overall survival in this malignancy.

## MATERIALS AND METHODS

### Culture and treatment of cell lines

Human ovarian cancer cell lines SKOV3 (HTB-77), ES-2 (CRL-1978), OV-90 (CRL-11732), TOV-21G (CRL-11730), TOV-112D(CRL-11731), breast cancer cell line MDA-MB-231 (HTB-26) and human embryonic kidney epithelial 293 cells (CRL-1573) were obtained from the American Type Culture Collection (Manassas, VA, USA). OVSAHO (JCRB 1046) was purchased from JCRB cell bank. MDAH2774 were purchased from AcceGen Biotechnology (Fairfield, NJ, USA). BR and BG1 cells were reported previously [43, 44]. The human endometrial cancer cell line ARK2 was kindly provided by Dr. Alessandro D. Santin (Yale University School of Medicine, New Haven, CT, USA). The immortalized human ovarian surface epithelial cell line (IOSE-80PC) was a gift from Dr. Peter CK Leung (British Columbia Children’s and Women’s Hospital, Vancouver, BC Canada) [45]. SKOV3, ES2, OV90, TOV21G, MDAH2774, TOV112D, BR, BG1 and 293 cells were cultured in DMEM/F12 culture medium, whereas ARK2, OVSAHO and IOSE-80PC were maintained in RPMI medium with 10% fetal bovine serum and appropriate amounts of penicillin and streptomycin. MDA-MB-231 was grown in L15 medium without 5% CO_2_ (with 10% fetal bovine serum and appropriate amounts of penicillin and streptomycin). For experiments with specific drugs, cells were pre-treated with 10 μM of the proteasome inhibitor MG132 (Sigma-Aldrich, St. Louis, MO, USA), 25 μM of the protein synthesis inhibitor cycloheximide (Sigma-Aldrich), the GSK3β inhibitor LY2090314 (Selleck Chemicals, Houston, TX, USA). Stably expressing wild-type or T265A/S269A/S273A ETS1 (ETS1-3A) 293 cells were transfected with Lenti-ETS1 or Lenti-ETS1 3A and treated with 5 μg/mL puromycin (Invitrogen, Carlsbad, CA, USA) at 48 h after transfection. After one month, individual clones were selected and assayed for ETS1 expression levels. All experiments were performed with mycoplasma-free cells.

### DNA construction

Full-length and truncated ETS1 constructs were amplified from ETS1 cDNA (NM_001143820, Sino Biological, Shanghai, China) and recombined into pNTAP (Agilent Technologies, Santa Clara, CA, USA) and pCMV-3xFlag (Agilent Technologies, Santa Clara, CA, USA) using an In-Fusion HD cloning kit (Clontech, Mountain View, CA, USA). ETS1 T265A/S269A/S273A mutants (ETS1-3A) and ETS1 S272A/S276A mutants (ETS1-2A) were generated by overlapping PCR with the Q5 Site-Directed Mutagenesis Kit (New England Biolabs, Ipswich, MA, USA). Halo-ETS1 and Lenti-ETS1 wild-type and mutant constructs were amplified from pCMV-3xFlag wild-type and mutant ETS1 and recombined into pFN21A (Promega; Halo-ETS1) or pLAS5w.PeGFP-I2-Puro (RNAi core laboratory, Academia Sinica, Taipei, Taiwan; Lenti-ETS1) vectors using the In-Fusion HD cloning kit. GSK3β truncated constructs and MMP9 promoter luciferase constructs have been previously described [31, 46]. The primers used for DNA constructs are shown in Supplementary Table 1. All constructs were finally confirmed with ABI autosequencer.

### Transfection of small interfering (si) RNA

SKOV3, MDAH2774 and 293 cells (5 × 10^5^ cells in a 6-well plate) were transfected with double-stranded RNA (50 nM) using the Lipofectamine RNAimax reagent (Invitrogen). GSK3β siRNA was from Santa Cruz (Santa Cruz Biotechnology). After 72 h of transfection, western blot was used to confirm silencing of the target genes.

### RNA extraction and real-time Q-PCR

Total RNA was isolated with the TRIzol reagent (Invitrogen). First-strand cDNA for RT-QPCR was synthesized with an oligo-T primer using the Superscript III first strand synthesis kit (Invitrogen). The SYBR green gene expression assay was used to investigate expression levels of *MMP-9, ETS1*; and *GSK3β*. The housekeeping gene *GAPDH* was used as an internal control. The primers used in the SYBR green gene expression assay are shown in Supplementary Table 1.

### Western blot analysis

The protocol used for western blot has been previously described in detail [47]. In brief, Cell lysates were prepared with RIPA buffer (150 mM NaCl, 20 mM Tris-Cl pH7.5, 1% Triton X-100, 1% NP40, 0.1% SDS, 0.5% deoxycholate) containing freshly added proteinase and phosphatase inhibitors (Bionovas, Toronto, Canada). Protein concentrations were measured with the Bradford method. Each sample (100 μg) was subjected to electrophoresis in 10% SDS-polyacrylamide gels and subsequently transferred onto nitrocellulose membranes. All experiments were conducted using commercially available antibodies and corresponding horseradish peroxidase-conjugated antibodies (Santa Cruz Biotechnology). The specific rabbit polyclonal antibody against phospho-ETS1-Thr265, Ser269 was produced by ABclonal (Woburn, MA, USA) using the following immunogen: CDRL(Tp)QSW(Sp)SQSS (phospho-ETS1-Thr265, Ser269). After immunization for five times in two months, the antibodies were purified by antigen affinity chromatography. Chemiluminescence reagents were obtained from Millipore. The signal intensity of the autoradiograms was quantified using the ImageJ software (https://imagej.nih.gov/ij/) after normalization for the corresponding actin intensity. The antibodies used for western blot are shown in Supplementary Table 2.

### Protein purification and *in vitro* kinase assay

Halo-tag ETS1 was purified with Halo Tag Protein purification system (Promega). In briefly, Halo-ETS1 transfected 293 cell pellets were lysed in Halo Tag purification buffer (1x PBS, 1mM DTT, 0.005% IGEPAL-CA630 (Sigma) and protease inhibitor). After freeze-thawing three times, the supernatants were added into HaloLink resin (Promega) according manufacturer instructions. Halo-ETS1 was released from the resin by using HaloTEV enzyme to cleavage TEV cutting site between Halo tag and ETS1 protein. The purified ETS1 protein was incubated with GSK3β (New England BioLabs) in GSK3 reaction buffer (20mM Tris-HCl, 10mM MgCl_2_, 2mM DTT and 200μM ATP) at 3° C for 30 mins. Finally, The phosphorylation levels of purified ETS1 were performed by western blot with anti-phosphoserine/threonine antibody (Cell signaling). The reagents and antibodies used for *in vitro* kinase assay are shown in Supplementary Table 2.

### Chromatin immunoprecipitation (ChIP) assay

The chromatin immunoprecipitation assay was performed as described previously [31]. In brief, Cells were treated with 1% formaldehyde at room temperature for 10 min to cross-link proteins to DNA. The reaction was stopped through the addition of glycine (0.125 M). Cells were scraped from culture dishes into PBS containing proteinase inhibitors. Pellets were resuspended in lysis buffer (5 mM PIPES/KOH pH 8.0, 85 mM KCL, 0.5% NP-40) and left on ice for 10 min. Nuclei collected by centrifugation were lysed with a nuclear lysis buffer (50 mM Tris pH 8.1, 10 mM EDTA, 1% SDS) and incubated on ice for 10 min. Lysates were sonicated to obtain chromatin having a mean length of approximately 600 bp. After centrifugation, 1 mg of protein supernatant was diluted 5-fold in ChIP dilution buffer (0.01% SDS, 1.1% Triton X-100, 1.2 mM EDTA, 16.7 mM Tris pH 8.1, 167 mM NaCl). Immunocomplexes were precipitated overnight at 4° C with 1 μg of specific antibodies, followed by four sequential washes with low salt, high salt, LiCl, and Tris-EDTA buffer (pH 7.8), respectively. The washed immunoprecipitate was then eluted in elution buffer (1% SDS, 0.1 M NaHCO3) at room temperature and subjected to two 15-min runs of vortex and centrifugation. Formaldehyde cross-links were reversed by incubation in a water bath at 65° C for 5 h. After treatment with proteinase K and subsequent phenol/chloroform extraction, DNA fragments were recovered by ethanol precipitation and amplified by QPCR on *MMP-9* promoter -166 region [31]. The primers and antibodies used for ChIP assay are shown in Supplementary Tables 1, 2.

### DNA transfection and luciferase reporter assay

The protocols for DNA transfection and luciferase reporter assays have been previously reported [48, 49]. Briefly, ovarian cancer cells (SKOV3 and MDAH2774) were trypsinized and re-suspended in serum-free RPMI medium (concentration: 10 million cells/mL). Cell suspensions (200 μL) were mixed with 5 μg DNA, transferred to a 2 mm-gap electroporation cuvette, and pulsed at 120 V for 70 msec with a BTX ECM2001 electroporator (Genetronics Inc., San Diego, CA, USA). Cells were subsequently re-seeded into a 6-well plate and maintained in DMEM/F12 with 10% fetal bovine serum for 24 h. ARK2 cells were transfected with Lipofectamine 2000 (Invitrogen) according to the manufacturer’s protocol. Finally, luciferase activity was measured using the Dual luciferase reporter assay system (Promega).

### Immunoprecipitation

Cell lysates were prepared using cell lysis buffer (20mM Tris-Cl pH7.4, 25mM NaCl and 0.1% NP40) containing proteinase inhibitors. Proteins (2 mg) were subjected to an overnight incubation commercially available resins or antibodies at 4° C under agitation. Corresponding resins were washed three times with wash buffer (20 mM Tris-Cl pH 7.4, 25 mM NaCl), boiled with sample buffer (0.25 M Tris-Cl pH 6.8, 0.08% SDS, 20% glycerol and 10% β-mercaptoethanol), electrophoresed (8% SDS-PAGE) and detected with specific antibodies or resins. The antibodies used for immunoprecipitation are shown in Supplementary Table 2.

### Transwell migration assay

Cell migration ability was assessed using a chemotaxis chamber (8μm pore, Corning, NY, USA). In brief, cells (1×10^5^ in 200 μL of serum free DMEM/F12) were loaded onto the upper part of the chamber, followed by the addition of serum-free DMEM/F12 (800 μL) containing 5 μg/mL fibronectin to the 24-well plate [50]. After insertion of the plate to the migration chamber, cells were passed through the polycarbonate membrane (pore size: 8 μm) located between the two chambers. Upon 24 h incubation at 37° C, the membrane was fixed in methanol for 10 min and stained in 20:2 water/Giemsa solution (Sigma-Aldrich) for 1 h. Cells that migrated onto the membrane were counted under a microscope.

### Wound closure assay

Wound closure assay was performed using a two-well migration insert with a defined cell-free gap (ibidi, Munich, Germany). Cells (density: 1 × 10^5^) were seeded overnight into the migration insert, which was subsequently removed to create the cell-free gap. Upon addition of serum-free medium, images of the cell-free gap were acquired at baseline and after 24 h of incubation. Cell-free gap areas were quantified with the ImageJ software (https://imagej.nih.gov/ij/) after normalization for the corresponding gap area at baseline.

### Immunohistochemistry

This study was approved by the institutional review board of Chang Gung Memorial Hospital, Linkou (IRB No. 201601560B0). Informed consent was waived for the retrospective nature of this study. Formalin-fixed, paraffin-embedded ovarian cancer specimens (46 serous adenocarcinomas and 29 endometrioid adenocarcinomas) were sectioned at 4 μm thickness and deparaffinized with xylene. Sections were dehydrated through a series of graded ethanol baths and stained with specific antibodies using an automated Leica Bond-MAX stainer (Leica Biosystem, Wetzlar, Germany). Expression levels of phospho-ETS1 and MMP9 were investigated by immunohistochemistry using anti-phospho-ETS1 Thr265/Ser269 antibody (custom-made; ABclonal Technology, Woburn, MA, USA) and MMP9 antibody (Abcam). Histoscores (0–300) were calculated as staining intensities (0–3) multiplied by the percentage of positively stained tumor cells (0–100%). The antibodies used for immunohistochemistry assay are shown in Supplementary Table 2.

### Protein digestion

Enriched protein extracts were reduced with dithiothreitol, alkylated with iodoacetamide, and digested with sequencing-grade trypsin (Promega). The tryptic digests were acidified with trifluoroacetic acid and desalted using Sep-Pak C18 cartridges (Waters Associates, Milford, MA, USA).

### Phosphopeptide enrichment

For phosphopeptide analysis, desalted peptide mixtures underwent further purification using TiO_2_ beads (5 μm, Titansphere; GL Sciences, Torrance, CA, USA). The bound fraction containing enriched phosphopeptides was eluted with 5% ammonium hydroxide solution containing 40% acetonitrile. Enriched phosphopeptides were vacuum-dried and re-suspended in 0.1% formic acid solution for subsequent nano-liquid chromatography/tandem mass spectrometry (nano LC-MS/MS).

### Tandem mass spectrometry-based protein identification

Tandem mass spectrometry was performed on Q Exactive^TM^ HF mass spectrometer (Thermo Fisher) coupled with an ACQUITY UPLC M-Class system (Waters Associates). Peptide mixtures were injected onto a trap column (Symmetry C18, 100 A, 5 μm, 180 μm × 20 mm, Waters Associates) connected with an analytic column (BEH C18 Column, 130 A, 1.7 μm, 75 μm × 250 mm, Waters Associates) and separated through a gradually increasing buffer B gradient (100% acetonitrile in 0.1% formic acid) over 75 min (flow rate: 260 nL/min). Peptide spectra were acquired in the positive ion mode (scan range: 375−1300 m/z). The ten most abundant precursor ions were dynamically selected for further fragmentation in the high-collision dissociation mode (with a normalized collision energy set at 28). The resolution of the full MS scan was set at 60000 with an m/z of 200 and an automatic gain control (AGC) of 3e6. The following parameters were used for the MS/MS scan: resolution, 15000; AGC target, 2e5; maximum injection time, 50 msec. The dynamic exclusion of the selected precursor ions was set between 20 and 30 sec.

### MS-based proteomic data analysis by proteome discoverer software and mascot search engine

The MS raw files were uploaded into the Proteome Discoverer (version 2.1, Thermo Scientific) software. The peak list for protein identification was generated using the Mascot search algorithm (version 2.5.1, Matrix Science, London, UK) against the Swiss-Prot human protein database. Carbamidomethylation of cysteine was set as a static or fixed modification. Oxidized methionine, phosphorylated serine or threonine, N-terminal glutamate conversion to pyroglutamate, and N-terminal acetylation were considered as dynamic modifications. Searches were done by allowing a single missed cleavage and with a mass accuracy set to 6 ppm and ±0.02 Da. In order to increase identification confidence, an overall false discovery rate below 1% was used. Moreover, only proteins with at least two unique peptides were accepted.

### Data analysis

Overall and recurrence-free survival curves were plotted with the Kaplan-Meier method. The optimal cutoff values for phospho-ETS1 histoscores were identified with the Youden’s index – with the goal of maximizing the sum of sensitivity and specificity to discriminate different events [51]. All calculations were performed with the SPSS 20.0 statistical package (SPSS Inc., Chicago, IL, USA). Two-tailed p values < 0.05 were considered statistically significant.

### *In vivo* animal model

The experimental protocols and animal care were approved by the animal care and use committee (IACUC) in Chang-Gung memorial hospital (IACUC No.2016113001). All tumor tissues were from our previous study [31]. In briefly, after 1x10^6^ murine ovarian surface epithelial cells (MOSEC cells) were injected to peritoneal cavity of C57BL/6 mice one week later, mice were intraperitoneally treated with vehicle control (20% Water:20% DMSO: 60% cromophore) or GSK3β inhibitor-LY2090314 (10 mg/kg) twice a week. One month later, mice were sacrificed and tumors in peritoneal cavity were collected for further detecting ETS1, MMP9 and actin with western blot.

## Supplementary Material

Supplementary Figures

Supplementary Tables
