# Supplementary material for: Glycogen synthase kinase-3 beta (GSK3β)-mediated phosphorylation of ETS1 promotes progression of ovarian carcinoma
Source: Aging (Albany NY). 2021 May 23;13(10):13739–63. doi: 10.18632/aging.202966 (PMC8202891; doi:10.18632/aging.202966)
Supplement: Supplementary Figures [file aging-13-202966-s001.pdf]

A Fragment ions of the double phosphorylated peptides

*p*T2*p*S6	L	pT	Q	S	W	pS	S	Q	S	S	F	N	S	L	Q	R
pS6pS10	L	T	Q	S	W	pS	S	Q	S	pS	F	N	S	L	Q	R
(y-ion)	16	15	14	13	12	11	10	9	8	7	6	5	4	3	2	1

B The MS/MS spectra of the double phosphorylated peptides

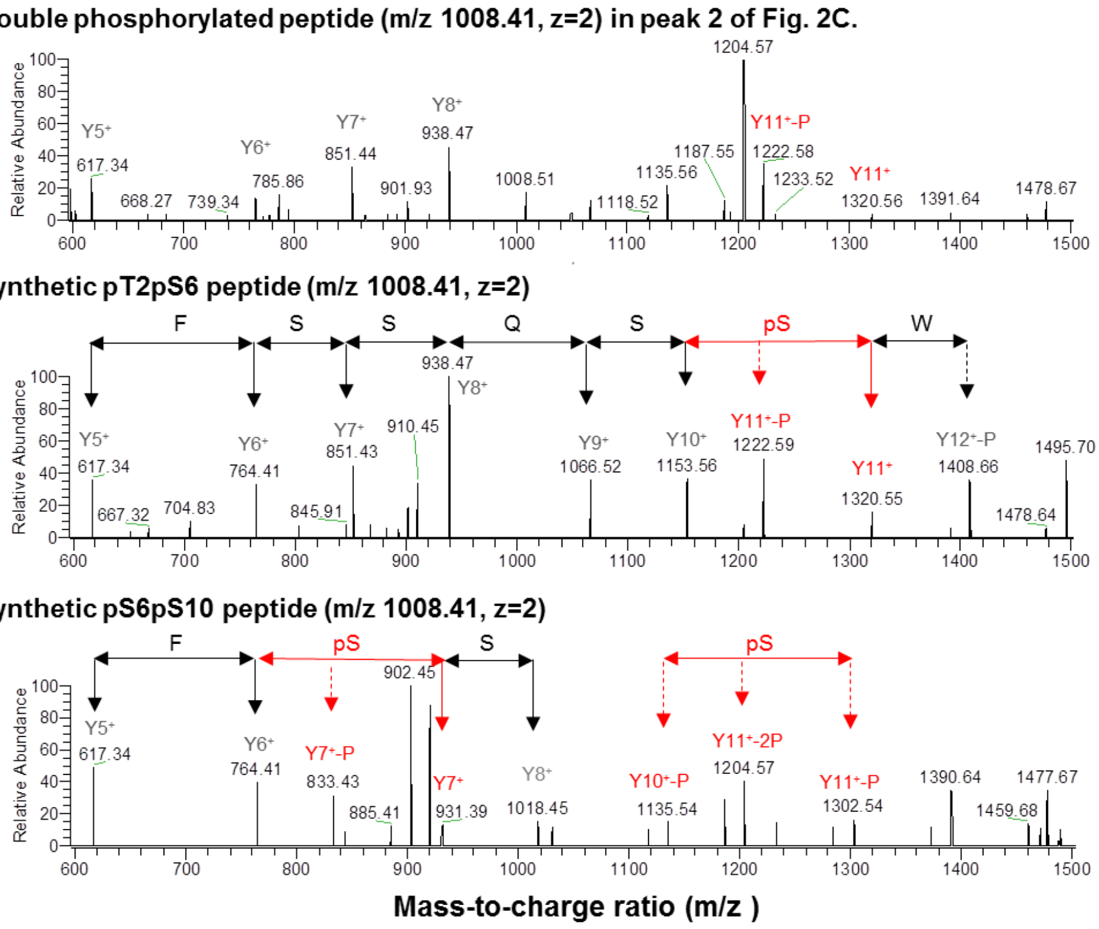

Supplementary Figure 1. Liquid chromatography-coupled tandem mass spectrometry (LC-MS/MS) identifies potentially phosphorylated amino acid residues. (**A**) The relationships between the fragmented Y-ion positions on the tandem mass spectrometry profile and two synthetic double phospho-peptides are shown in the Table. The direction of the Y-ion position started from the end of the peptide C-terminus. Phosphorylated residues of the two synthetic peptides (pT2S6 and pS6S10) are shown in red and correspond to the Y11 and Y15 fragment ions for pT2S6 and to the Y7 and Y11 fragment ions for pS6S10. (**B**) The sequence and phosphorylation sites of the potential GSK3β-catalyzed double-phosphorylation event (peak2 in Figure 2C) was analyzed with LC-MS/MS (upper panel). Using two synthetic peptides (pT2S6 and pS6S10) as reference standards, the phosphorylated peptide in peak2 displayed the first phosphorylation event at Y11 (upper panel, red) but not at Y7. The pT2S6 peptide exhibited the first phosphorylation event at Y11 (middle panel, red). However, the pS6S10 peptide was characterized by a first phosphorylation event at Y7, followed by a second phosphorylation at Y11 (lower panel, red). The fragment ion spectra of the candidate for GSK3β-catalyzed phosphorylation were remarkably more similar to the pT2pS6 fragmentation profile – without phosphorylation ions being evident between the y5 and y8 fragments.

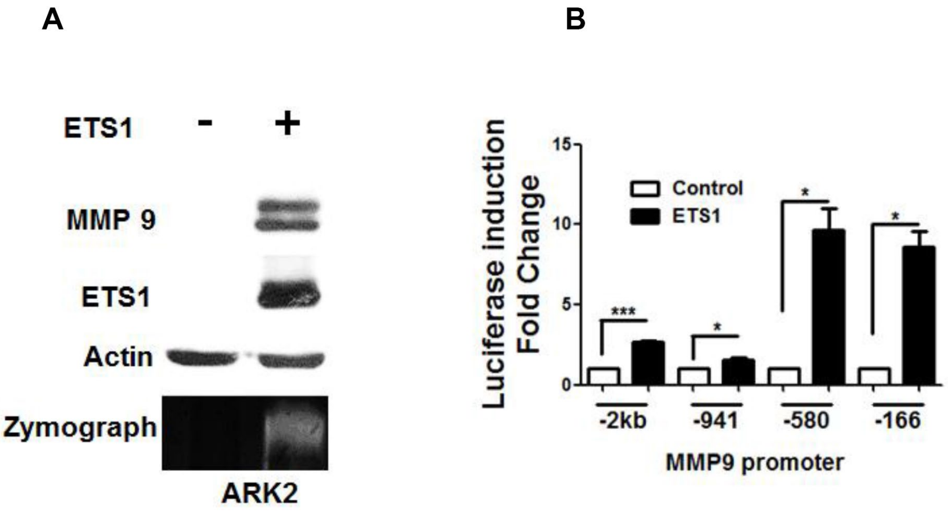

Supplementary Figure 2. ETS1 activates MMP-9 transcription. (**A**) Overexpression of ETS1 in ARK2 cells induced MMP-9 protein expression and activity. Flag-ETS1, endogenous MMP-9, and actin were analyzed with western blot using anti-Flag, anti-MMP-9 and anti-actin antibodies. Endogenous actin served as a loading control. The zymograph (lower panel) revealed an increased MMP-9 activity in ETS1-overexpressing cells. (**B**) ETS1 activated different truncated MMP-9 promoter constructs. Luciferase activities were measured at 24 h after co-transfection of ETS1 and distinct truncated MMP-9 promoter constructs in ARK2 cells. The MMP-9 promoter activity measured in transfected Flag-control cells was set as 1. Results are means ± standard errors of the mean from three independent experiments. $^*p < 0.05$, $^{***}p < 0.001$.

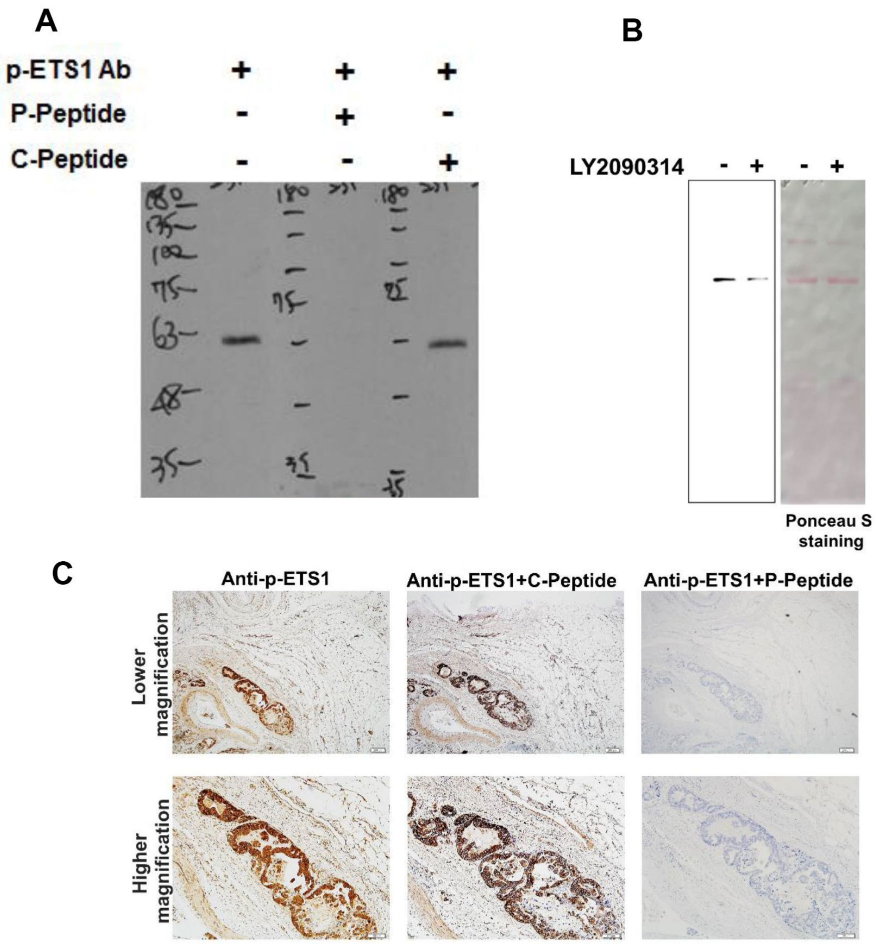

Supplementary Figure 3. The anti-phospho-ETS1 antibody specifically detects the endogenous target in cell line and tissues. (**A**) The anti-phospho-ETS1 antibody was capable of detecting 50 μg of whole-cell lysate from MDA-MB-231 cells in the absence of phospho-ETS1 peptide (p-peptide; left panel), in presence of p-peptide (middle panel) or when the non-phospho-ETS1 peptide (c-peptide, right panel) was used to neutralize the antibody. (**B**) Phosphorylation status of purified Halo tagged ETS1 proteins overexpressed in 293 cells in presence or absence of GSK3B inhibitor-LY2090314 for 12 hours was verified with anti-phospho-ETS1 antibody. Ponceau S staining (right panel) was shown as western blot loading control. (**C**) Immunohistochemistry was used to analyze the expression of phospho-ETS1 in ovarian cancer tissues. Experiments were performed with the anti-phospho-ETS1 antibody alone (left panel), the anti-phospho-ETS1 antibody pre-incubated with non-phospho-ETS1 peptide (c-peptide, middle panel), and with the anti-phospho-ETS1 antibody neutralized with the phospho-ETS1 peptide (p-peptide, right panel).

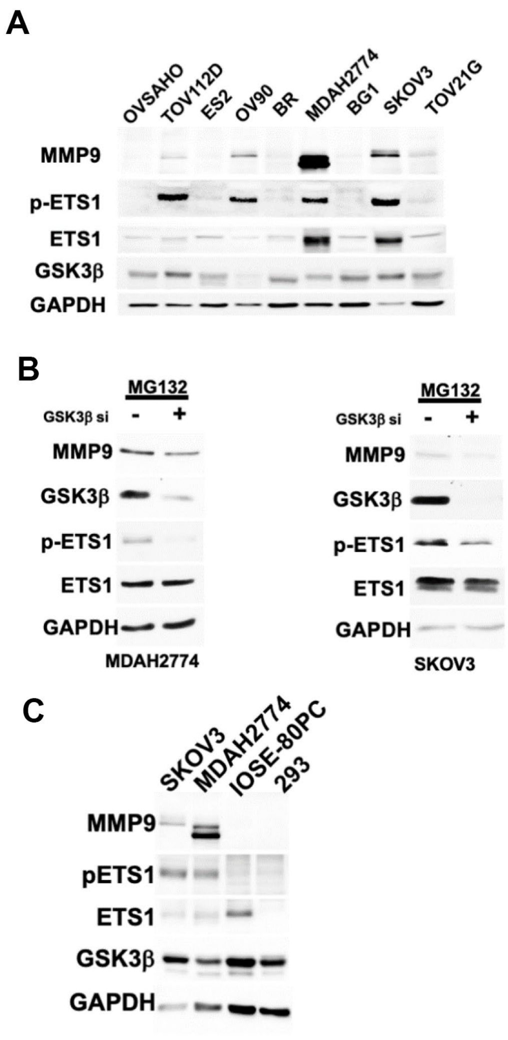

Supplementary Figure 4. Phospho-ETS1 is expressed in ovarian cancer and normal cell lines. (**A**) Western blot was used to analyze the expression levels of endogenous MMP-9, phospho-ETS1, total ETS1, GSK3β and GAPDH in nine different ovarian cancer cell lines. (**B**) Knockdown of GSK3β with siRNA inhibited phospho-ETS1 and MMP-9 expression in both MDAH2774 cells (left panel) and SKOV3 cells (right panel) treated with MG132 (10 μM). (**C**). The expression levels of MMP9, phospho-ETS1 (pETS1), total ETS1 (ETS1) and GSK3β in two ovarian cancer cell lines (SKOV3 and MDAH2774) and two normal cell lines (IOSE-80PC and 293).
