# Supplementary material for: Glycogen synthase kinase-3 beta (GSK3β)-mediated phosphorylation of ETS1 promotes progression of ovarian carcinoma
Source: Aging (Albany NY). 2021 May 23;13(10):13739–63. doi: 10.18632/aging.202966 (PMC8202891; doi:10.18632/aging.202966)
Supplement: Supplementary Tables [file aging-13-202966-s002.pdf]

SUPPLEMENTARY TABLES

Supplementary Table 1. Primer sequences used for DNA construction and QPCR.

Primer	Sequence
ETS1 forward	5'-CTGCCCGGGCGGATCCATGAGCTACTTTGTGGATTCTGC-3'
ETS1 reverse	5'-CGGTATCGATAAGCTTTCACTCGTCGGCATCTGG-3'
ETS1 1−378 reverse	5'-CGGTATCGATAAGCTTTGGTCCACTGCCTGTGTAGC-3'
ETS1 1−197 reverse	5'-CGGTATCGATAAGCTTGCGGGATTCTGGATAGGC-3'
ETS1 191−486 forward	5'-CTGCCCGGGCGGATCCATGGCCTATCCAGAATCCCGC-3'
ETS1 363−486 forward	5'-CTGCCCGGGCGGATCCATGGTCATTCCTGCTGCTGCC-3'
Halo-ETS1 forward	5'-CAGAGCGATAACGCGATCGCCATGAGCTACTTTGTGGATTCTGC-3'
Halo-ETS1 reverse	5'-AGCCCGAATTCGTTTAAACTCACTCGTCGGCATCTGG-3'
Lenti-ETS1 forward	5'-TCGAACCTTAGGGATATCATTAACCCTCACTAAAGGGA-3'
Lenti-ETS1 reverse	5'-TCAAGATCTAGAATTCTAATACGACTCACTATAGGG-3'
ETS1 T265A/S269A/S273A mutants (ETS1-3A) forward	5'-CAGCCAGTCAGCTTTCAACAGCCTGCAGCGT-3'
ETS1 T265A/S269A/S273A mutants (ETS1-3A) reverse	5'-GCCCAGGACTGGGCGAGGCGATCACAACTATCG-3'
ETS1 S272A/S276A mutants (ETS1-2A) forward	**5'**-CAACGCCCTGCAGCGTGTTCCCTCC-3'
ETS1 S272A/S276A mutants (ETS1-2A) reverse	**5'**-AAAGATGCCTGGCTGCTCCAGGACTG-3'
MMP9 QPCR forward	5'-CTTCACTTTCCTGGGTAA-3'
MMP9 QPCR reverse	5'-ACAAACTGTATCCTTGGT-3'
ETS1 QPCR forward	5'-GACCTAGCAACACTTATG-3'
ETS1 QPCR reverse	5'-CACATTCCATATCTGAGAC-3'
GSK3β QPCR forward	5'-GAGGTCTATCTTAATCTG-3'
GSK3β QPCR reverse	5'-GATACATATACAACTTGAC-3'
GAPDH QPCR forward	5'-GGTATCGTGGAAGGACTCATGAC-3'
GAPDH QPCR reverse	5'-ATGCCAGTGAGCTTCCCGT-3'
MMP9 ChIP assay -5F	5'-GCAGTTGAAGAATCCTAA-3'
MMP9 ChIP assay -5R	5'-CATTCCTGTAATCTTAGCA-3'

Supplementary Table 2. Antibodies and immunoprecipitation assay reagents.

Reagent	Source	Identifier
Immunoprecipitation and chromatin immunoprecipitation assay		
Halo tag resin (IP, ChIP)	Promega	Cat#G1915
Streptavidin beads (CBP tag, IP)	Thermo Fisher	Cat#15942-050
Protein G beads (IP)	Millipore	Cat#16-266
Antibody		
ETS1 (IP, WB, IF)	Cell Signaling	Cat#14069
MMP9 (WB, IHC)	Abcam	Cat#ab76003
Calmodulin binding peptide tag (CBP tag, WB)	Millipore	Cat#07-482
Halo tag (WB)	Promega	Cat#G9211
GSK3β (IP, WB, IF)	Cell Signaling	Cat#9315
STIP1 (WB)	Genetex	Cat#GTX103068
GAPDH (WB)	Santa Cruz	Cat#sc-32233
HA tag (WB)	Cell Signaling	Cat#3724
Ubiquitin (WB)	Cell Signaling	Cat#3936
p300 (IP)	Abcam	Cat#ab14984
Flag tag (IP, WB)	Sigma-Aldrich	Cat#F1804
phospho-serine/threonine (IP)	Cell Signaling	Cat#9631
phospho-ETS1-Thr265, Ser269 (WB, IHC)	ABclonal	Custom made

Abbreviations: IP: Immunoprecipitation, ChIP: Chromatin immunoprecipitation, WB: Western blot, IF: Immunofluorescence, IHC: Immunohistochemistry.
